# Pharmacological and non-pharmacological treatments for irritable bowel syndrome: Protocol for a systematic review and network meta-analysis

**DOI:** 10.1097/MD.0000000000016446

**Published:** 2019-07-26

**Authors:** Shenghui Zhou, Xiaoli Liu, Xiaoxia Wang, Fenglin Xi, Xiaoke Luo, Liang Yao, Hao Tang

**Affiliations:** a College of Pharmacy, Ningxia Medical University, Yinchuan, Ningxia Hui Autonomous Region; b Department of Pharmacy, Baiyin Central Hospital, Baiyin; c Gansu Gem Flower Hospital, Lanzhou, Gansu; d Department of Pharmacy, Gansu Provincial People's Hospital, Lanzhou, PR China.

**Keywords:** IBS, irritable bowel syndrome, network meta-analysis, systematic review

## Abstract

**Background:**

The global prevalence of Irritable bowel syndrome (IBS) is estimated to be as high as 15% and a number of different non-pharmacological and pharmacological treatments have been used to manage IBS in clinical practice, which poses great challenges for clinicians to make appropriate decisions. Hence, a systematic review and network meta-analysis on all available pharmacological and non-pharmacological treatments for IBS is needed to provide reliable evidence.

**Methods:**

We will search the Cochrane Central Register of Controlled Trials (CENTRAL), the Cochrane IBD Group Specialized Trials Register, MEDLINE, EMBASE, and Chinese Biomedical medicine (CBM) from inception to 31, May 2019. Randomized controlled trials of pharmacological and nonpharmacological interventions for IBS will be included. Study quality will be assessed on the basis of the methodology and categories described in the Cochrane Collaboration Handbook. Primary outcomes are global or clinical improvement and quality of life. A Bayesian network meta-analysis would be performed, and relative ranking of agents would be assessed. A node splitting method will be used to examine the inconsistency between direct and indirect comparisons when a loop connecting 3 arms exists.

**Results:**

Researchers will rank the effectiveness and safety of the potentials interventions for IBS according the characteristics of patients by conducting an advanced network meta-analysis based on Bayesian statistical model, and interpret the results by using GRADE approach.

**Conclusion:**

The conclusion of our study will provide updated evidence to rank the effectiveness and safety of pharmacological and non-pharmacological interventions for IBS.

**Ethics and dissemination:**

Ethical approval is not applicable since this study is a network meta-analysis based on published trials.

**Protocol registration number:**

CRD42018083844.

## Abstract 2

Supplemental Digital Content is available in the text

## 1 Introduction

### 1.1 Description of the condition

Irritable bowel syndrome (IBS) is a functional gastrointestinal disorder characterized by chronic and recurrent abdominal pain and alterations in stool consistency or frequency but without gross abnormalities. Currently, the global prevalence of IBS is estimated to be as high as 15%,^[1]^ and it is estimated that IBS has a prevalence of approximately 10% to 20% in western countries.^[2]^ A combination of characteristic symptoms and the absence of warning signs on examination is used for IBS diagnosis, and the well-accepted Rome criteria, now is in its forth version.^[3]^ IBS is commonly classified into 4 main subtypes, that is, diarrhea predominant (IBS-D), constipation predominant (IBS-C), mixed (IBS-M), and unclassified (IBS-U). Moreover, IBS patients can also be divided into 2 categories, namely nonspecific and post-infectious (PI-IBS).

As a multifactorial disease, the pathophysiology of IBS is still inadequately clarified. Diverse factors, such as genes, lifestyle and diets, psychosocial factors, brain-gut axis dysfunction, intestinal inflammation, intestinal microbiota alteration, as well as intestinal immune disruption, are all considered to play important roles in the pathogenesis of IBS,^[4]^ thus there are not very satisfactory treatments for IBS patients. Currently, most of managements of IBS primarily aim at symptoms relief, that is, laxatives for constipation, antispasmodics for pain, anti-motility drugs for diarrhea, and antidepressants for mood and physical activity.^[5]^ The multiple and persistent symptoms of IBS contribute to high work absenteeism, high socioeconomic burden, and decline of life quality. IBS has been estimated to be the cause of between 8.5 and 21.6 days off work per year,^[6,7]^ and the chronicity of IBS symptoms leads to increased use of secondary health care services.

### 1.2 Description of the interventions

A number of different non-pharmacological and pharmacological treatments have been used to manage IBS in clinical practice, which lead to great challenges for clinicians to make appropriate decisions.

#### 1.2.1 Lifestyle and dietary interventions

Lifestyle and diet interventions sometimes are considered before pharmacological treatment. Lack of exercise, food deficiencies, lack or excess of dietary fiber intake, and lack of suitable times for defecation could contribute to the development of IBS, specifically constipation-predominant IBS.^[8]^ Thus, an increase in dietary fibers and regular exercise might benefit constipated IBS patients.^[9]^ Excessive caffeine consumption, indigestible carbohydrates and high lactose intake have been found to contribute to diarrhea-predominant IBS.^[10,11]^ Thus a stepwise food exclusion approach might be effective if the symptoms are mild to moderate.^[12]^ The evaluation of probiotics to treat IBS has been summarized in meta-analytic studies that showed modest improvements for bloating, abdominal pain and bowel movement difficulties. No specific probiotic strain was found to be superior to another.^[9,13]^

#### 1.2.2 Psychotherapy interventions

The impact of various forms of psychotherapy (e.g., cognitive behavioral therapy, dynamic psychotherapy, hypnotherapy, biofeedback, and relaxation therapy) on IBS has been evaluated. According to guidelines of the National Institute for Health and Care Excellence (NICE),^[14]^ British Society of Gastroenterology^[15]^ and the American Gastroenterology Association,^[16]^ psychotherapeutic interventions are usually reserved for severe forms of IBS that show high incidence of a comorbid psychological disorder^[17]^ or if a known comorbidity with a depressive or anxiety disorder exists. The hypnotherapy and stress management over the course of 6 weeks to 6 months in patients with IBS-D or IBS-M seem to be effective to improve symptoms.^[18]^ Concomitant treatment of diagnosed depression or anxiety disorders through psychotherapy and pharmacological treatment often helps to alleviate specific IBS symptoms.^[16]^

A published meta-analysis and systematic review showed that the heterogeneity of psychotherapeutic treatment results in a 25% chance that a patient will benefit from any type of psychotherapy,^[19]^ while hypnotherapy and stress management had a higher rate of success with 52% and 67%, respectively.^[20,21]^ a Cochrane review based on 3 RCTs concluded 68% of patients in the homeopathy group got symptoms improved compared to 52% of placebo.^[22]^

#### 1.2.3 Pharmacological treatment

After lifestyle and diet changes have failed to alleviate or resolve IBS symptoms, the most common treatment approach is pharmacotherapy.

Prokinetics are used to enhance intestinal contractions and facilitate the movement of fecal matter by acting as dopamine antagonists, 5-HT3 antagonists and/or 5-HT4 agonists. Despite inconsistent benefits to IBS-C patients, they are widely used and increase GI motility with concomitant increase in secretory activity and effects as visceral analgesics.^[22]^ Tegaserod is the only prokinetic drug approved by the US Food and Drug Administration for the treatment of IBS, but it was restricted in 2007 due to risk of cardiovascular ischemic events.^[23]^ laxative lubiprostone was approved in 2008 for treatment of IBS-C in women, which acts as a chloride channel activator that increases water secretion into the feces.^[24,25]^

The anticholinergic antispasmodics are frequently used to reduce abdominal pain, visceral sensitivity and GI motility. Whereas unspecific anticholinergics such as hyoscine or pinaverium are used to treat both IBS-C and IBS-D, specific muscarinic M3 receptor antagonists such as darifenacin and zamifenacin might provide a more specific treatment approach.^[26,27]^ A meta-analysis of clinical trials with antispasmodics revealed that the clinical benefit of cimetropium, pinaverium, hyoscine and otilonium was highest whereas studies with pirenzepine and propinox favored the placebo treatment over the actual drug.^[28]^ As expected with the anticholinergic antispasmodics, the most common adverse effects were dry mouth, dizziness and blurred vision. In addition, antispasmodics will reduce GI motility and therefore need to be given in conjunction with a prokinetic or laxative in order to increase GI motility.

Opioid agents and anticholinergic agents are commonly used pharmacological treatments for IBS-D. Loperamide is an opioid agonist that acts on m-receptors of the myenteric plexus in the large intestines without being absorbed or causing CNS effects after oral administration.^[29]^ Loperamide, commonly used for short-term diarrhea due to bacterial GI infections, should only be given in low doses as needed to patients with IBS-D. Ondansetron, granisetron, alosetron and cilansetron are all selective 5-HT3 receptor antagonists frequently prescribed for IBS-D as well as for other conditions such as vomiting, and nausea associated with chemotherapy.^[30]^

#### 1.2.4 Complementary and alternative interventions

World Gastroenterology Organisation Global Guidelines for IBS^[31]^ mentions that complementary and alternative therapies have been used continually and reported benefit in persons with IBS although the effectiveness of the therapies has not been clinically well studied. A Cochrane review of herbal medicines for the treatment of IBS^[32]^ identified several well-designed clinical studies that showed improvement of IBS symptoms. One study employing a variety of Chinese herbal medicines, given alone or in a fixed combination, showed significant improvement of various IBS symptoms over a placebo treatment that extended beyond the end of the study.^[33]^

Other alternative treatments frequently used by patients suffering from IBS are peppermint oil and acupuncture. The use of peppermint oil has been evaluated through 2 meta-analysis studies that compared clinical trials of peppermint oil preparations with a placebo.^[28,34]^

Acupuncture, which has been used as a therapeutic treatment in Chinese traditional medicine for centuries, has gained significant attention over the past decades in Western medicine. A Cochrane reviews of a few small clinical trials involving the effect of acupuncture treatment in patients with IBS included only studies that used actual acupuncture versus sham acupuncture, any other active interventions, or no treatment (negative control) to alleviate IBS symptoms.^[35]^ The meta-analysis revealed that the effects of acupuncture on IBS symptoms were variable and did not differ significantly from the sham acupuncture treatment or any other interventions.^[36]^ This may be due to inconsistencies in study designs and possible inclusion of patients who were not thoroughly diagnosed with IBS prior to treatment.

In general terms, the numerous alternative options exist for IBS in clinical practice. However, the reliability of these evidence varied greatly, some of them based on outdated SRs which just included small sample size and concluded imprecise and different results, and some others just based on RCTs without evaluation of risk of bias, all these discrepancies could lead to obstacles and challenges for physicians to give optimal management for IBS, thus a comprehensive comparisons is necessary to rank all available pharmacological and non-pharmacological interventions and their combinations based on effectiveness and safety outcomes. The comprehensive comparisons should include direction comparisons and indirection comparisons of available RCTs, that is, conducting a Network meta-analysis (NMA).

## 2 Methods

### 2.1 Design and registration

This systematic review will be reported in accordance with the Preferred Reporting Items for Systematic Review and Meta-Analyses (PRISMA) Statement and is registered in the PROSPERO database (International Prospective Register of Systematic Reviews) under the number CRD42018083844. No ethical approval is required since this study used data already in the public domain.

### 2.2 Data sources

We will search the following databases from inception to May 2019 to identify studies: the Cochrane Central Register of Controlled Trials (CENTRAL), the Cochrane IBD Group Specialized Trials Register, MEDLINE, EMBASE, and Chinese Biomedical medicine (CBM). The search strategies will be customized for each database and we will use recommended Cochrane search string for the identification of RCTs. Detailed search strategy could be found in the appendix.

### 2.3 Study selection

We use ENDNOTE X7 literature management software to screen and manage citations yield from electronic databases. Pilot tests will be performed for literature screening and data extraction, and remarks will be made to ensure high inter-rater reliability among the reviewers. Study eligibility will be assessed in 2 stages. First, pairs of reviewers will independently examine the titles and abstracts in ENDNOTE to identify related studies. Then, each full text article from the screening stage will be obtained and evaluated. Excluded trials and the reasons will be recorded and any disagreement will be resolved through discussion or consultation with an independent third adjudicator. The criteria of including studies are as follows:

1) Adults and children with a diagnosis of IBS based on diagnostic criteria including Rome I, Rome II or Rome III or Rome IV will be included. Appropriate participants will be included regardless of gender, race, educational status or duration of IBS.
2) Randomized controlled trials (RCTs) of pharmacological and non-pharmacological treatments for IBS will be considered for inclusion regardless of publication status and language of publication. Studies using active, no treatment, sham treatment, or placebo controls will be included. Trials with quasi-random designs will not be considered for inclusion.
3) At least one of the following outcomes should be included, primary outcomes included: i) global or clinical improvement as defined by the included studies (e.g., IBS Severity Scoring System (IBS-SSS), or the Gastrointestinal Symptom Rating Scale (GSRS)). However, various instruments are available to measure health- related outcomes in IBS, and the quality of these scales varied and some may be not validated. This may associate with bias, therefore we will just include studies that used the published and validated scales. ii) Quality of life measured by a validated quality of life scale (e.g., overall well-being, IBS Quality of Life questionnaire (IBS-QoL), Short Form Health Survey (SF36). Secondary outcomes will include: i) Adverse events; ii) withdrawal due to adverse events; iii) stool frequency; iv) stool consistency (e.g. rated by the Bristol Stool Scale); v) improvement in abdominal pain frequency and severity; vi) depression; vii) anxiety.

### 2.4 Data extraction

Data abstraction will be completed by independent pairs of reviewers after pilot-testing of the data extraction form. Interventions were coded independently by a clinician and a methodologist using a pre-established coding. Included interventions were classified into the following 2 categories: pharmacological interventions and non-pharmacological interventions. Furthermore, 2 independent reviewers will extract the data of interest. All the discrepancies will be resolved through discussion or a third reviewer. The following descriptive data from eligible studies will be abstracted: country of origin, year of publication, disease course, interventions, treatment schema and doses, number of participants, patient characteristics, background therapies, outcomes measurement or monitoring, length of follow-up, definition and data of primary outcome.

### 2.5 Assessment of risk of bias of included studies

Two reviewers will evaluate the risk of bias of the selected RCTs according to the criteria and technique proposed in the Cochrane Handbook V.5.2.0,^[37]^ which includes random sequence generation (selection bias), allocation concealment (selection bias), blinding of participants and personnel (performance bias), blinding of outcome assessment (detection bias), incomplete outcome data (attrition bias), selective reporting (reporting bias) and other bias. Each study will be assigned a level of risk of bias (high risk, unclear risk, low risk) for each item. Any disagreement will be resolved through discussion or consultation with an independent third adjudicator.

### 2.6 Assessment of the quality of evidence

Using the Grading of Recommendations Assessment, Development and Evaluation (GRADE), the quality of evidence will be evaluated as 4 levels—high quality, moderate quality, low quality and very low quality.^[38]^ This process will be performed with the online guideline development tool (GDT, http://gdt.guidelinedevelopment.org/)

### 2.7 Data processing and analysis

#### 2.7.1 Geometry of the network

A network plot will be drawn to present the geometry of the network of comparisons across trials to ensure an NMA is feasible. Trials will be excluded if they are not connected by interventions. Nodes in network geometry represent different interventions and edges represent head to head comparisons. The size of nodes and thickness of edges are associated with sample sizes and numbers of RCTs, respectively.

#### 2.7.2 Pairwise meta-analysis

Pairwise meta-analyses will be performed using Review Manager 5.3.3 (Cochrane Collaboration, Denmark). Odds ratios (ORs) with 95% CI will be used for dichotomous outcomes. Mean differences (MDs) or standard mean differences (SMDs) with 95% CI will be used for continuous outcomes. We will assess clinical and methodological heterogeneity through examination of the characteristics of the included trials. Heterogeneity across trials will be assessed by c^2^ and I^2^ statistics. Pairwise random-effects meta-analysis will be used to pool the results.^[37]^ Publication bias will be examined using Begg and Egger funnel plot method when applicable.^[38,39]^ In addition, the contour-enhanced funnel plot will be obtained as an aid to distinguish asymmetry due to publication bias.^[40]^

#### 2.7.3 Network meta-analysis

We will perform Bayesian NMAs with the package ‘gemtc’ V.0.8.1 of R-3.3.2 software^[41]^ to compare the effects of different prophylactic agents. The Markov Chains Monte Carlo sampler will be used to generate samples. A total of 5000 simulations for each chain will be set as the ‘burn-in’ period. Then, posterior summaries will be based on 100,000 subsequent simulations. Model convergence will be assessed using the Brooks–Gelman–Rubin plots method.^[42]^ Global heterogeneity will be assessed on the bias of the magnitude of heterogeneity variance parameter (I^2^ or τ^2^) estimated from the NMA models using the mtc anohe command of the ‘gemtc’ package. A node splitting method will be used to examine the inconsistency between direct and indirect comparisons when a loop connecting 3 arms exists.^[43]^ The ranking probabilities for all treatments will be estimated, and a treatment hierarchy using the probability of being the best treatment can be obtained. This process will be performed using the cumulative ranking curve (SUCRA).^[44]^ SUCRA values are expressed as percentages—100% for the best treatment, 0% for the worst treatment.^[44]^ We will also try to use the frequentist approach to compare stability if necessary.^[45,46]^

## 3 Discussion

The purpose of this project is to provide reliable evidence of pharmacological and non-pharmacological treatments for IBS. Researchers will rank the effectiveness and safety of the potentials interventions for IBS according the characteristics of patients by conducting an advanced network meta-analysis based on Bayesian statistical model. In order to promote clinical practice, researchers will cooperate with the guideline development panel to translate the findings of NMA into recommendations in the future IBS guidelines.

## Acknowledgments

We would like to acknowledge Editage company for polishing the language.

## Author contributions

ZS, LXL, WXX, and TH contributed to the conceptualization, study design, search strategy, protocol development, and review by revising different versions. ZS, LXL, WXX, XF, LXK and TH were involved in the supervision, ensured the absence of errors, and arbitrated in case of disagreement. ZS and TH engaged in the manuscript writing and analysis. All authors have read and approved the final version of the manuscript.

**Conceptualization:** Xiaoli Liu, Xiaoxia Wang, Fenglin Xi, Liang Yao, tang hao.

**Data curation:** Xiaoli Liu, Liang Yao.

**Formal analysis:** Shenghui Zhou.

**Methodology:** Shenghui Zhou, tang hao.

**Project administration:** Shenghui Zhou.

**Supervision:** tang hao.

**Writing – original draft:** Shenghui Zhou.

**Writing – review & editing:** Xiaoli Liu, Xiaoxia Wang, Fenglin Xi, Xiaoke Luo, tang hao.

## Supplementary Material

Supplemental Digital Content
