# Supplementary material for: Pharmacological and non-pharmacological treatments for irritable bowel syndrome: Protocol for a systematic review and network meta-analysis
Source: Medicine (Baltimore). 2019 Jul 26;98(30):e16446. doi: 10.1097/MD.0000000000016446 (PMC6709000; doi:10.1097/MD.0000000000016446)
Supplement: Supplemental Digital Content [file medi-98-e16446-s001.docx]

| Box detailed search strategy  MEDLINE  1. random$.tw  2. factorial$.tw  3. (crossover$ or cross over$ or cross-over$).tw  4. placebo$.tw  5. single blind.mp  6. double blind.mp  7. triple blind.mp  8. (singl$ adj blind$).tw  9. (double$ adj blind$).tw  10. (tripl$ adj blind$).tw  11. assign$.tw  12. allocat$.tw  13. crossover procedure/  14. double blind procedure/  15. single blind procedure/  16. triple blind procedure/  17. randomized controlled trial/  18. or/1-17  19. (exp animal/ or animal.hw. or nonhuman/) not (exp human/ or human cell/ or (human or humans).ti.)  Irritable bowel syndrome.mp  20. 18 not 19  21 Colonic Diseases, Functional.mp 22 “irritable bowel syndrome”.tw 23 irritable bowel syndrome*.tw 24 IBS.tw 25 “functional abdominal pain”.tw 26 “functional gastrointestinal disorders”.tw 27 or/21-26  28.20 and 27  **Cochrane Central Register of Controlled Trials (CENTRAL)**  1. MeSH descriptor: [Irritable Bowel Syndrome] explode all trees 2. MeSH descriptor: [Colonic Diseases, Functional] explode all trees 3. “irritable bowel syndrome”:ti,ab,kw  4. irritable bowel syndrome*:ti,ab,kw  5. IBS:ti,ab,kw (Word variations have been searched) 6. “functional abdominal pain”:ti,ab,kw  7. “functional gastrointestinal disorders”:ti,ab,kw  8. or/1-7  **EMBASE**  1. random$.tw.  2. factorial$.tw.  3. (crossover$ or cross over$ or cross-over$).tw.  4. placebo$.tw.  5. single blind.mp.  6. double blind.mp.  7. triple blind.mp.  8. (singl$ adj blind$).tw.  9. (double$ adj blind$).tw.  10. (tripl$ adj blind$).tw.  11. assign$.tw.  12. allocat$.tw.  13. crossover procedure/  14. double blind procedure/  15. single blind procedure/  16. triple blind procedure/  17. randomized controlled trial/  18. or/1-17  19. (exp animal/ or animal.hw. or nonhuman/) not (exp human/ or human cell/ or (human or humans).ti.)  20. 18 not 19  21. ‘intestine function disorder’/exp  22. ‘irritable colon’/  23. ‘irritable bowel syndrome*’/  24. IBS/  25. ‘functional gastrointestinal disorder*’/  26. ‘functional abdominal pain’/  27. or/21-26  28. 20 and 27  **CBM**  1. “肠易激综合征” [不加权:扩展]  2.“IBS”[常用字段:智能]  3. or/1-2  4. “随机” [常用字段:智能]  5. 3 and 4 |
| --- |
